# Subjective memory complaints and memory performance in patients with borderline personality disorder

**DOI:** 10.1186/s12888-014-0255-2

**Published:** 2014-09-06

**Authors:** Thomas Beblo, Christoph Mensebach, Katja Wingenfeld, Nina Rullkoetter, Nicole Schlosser, Martin Driessen

**Affiliations:** Department of Research, Clinic of Psychiatry and Psychotherapy Bethel, Ev. Hospital Bielefeld, Remterweg 69-71, 33617 Bielefeld, Germany; Department of Psychology, University of Bielefeld, Bielefeld, Germany; Department of Psychosomatic Medicine and Psychotherapy, University Duisburg-Essen, Essen, Germany; Department of Psychiatry, Charité University Berlin, Campus Benjamin Franklin, Berlin, Germany

**Keywords:** Borderline Personality Disorder (BPD), Memory, Complaints, Tests

## Abstract

**Background:**

It is still a matter of debate as to whether patients with Borderline Personality Disorder (BPD) suffer from memory deficits. Existing studies indicate no or small impairments in memory test performance. However, it was shown in patients with related disorders, such as depression, that self-reported impairment exceeds test malfunction. In the present study we assessed memory performance of BPD patients through the use of memory tests and a questionnaire for subjective memory complaints (SMC) in everyday life.

**Methods:**

Thirty-two patients with BPD and 32 healthy control subjects were included in the study. The groups of subjects were comparable with respect to age, education, and gender. Subjects completed verbal and nonverbal memory tests, as well as the everyday memory questionnaire (EMQ).

**Results:**

BPD patients reported severe SMC but did not show memory test impairment. The results remained stable even when all BPD patients with acute or lifetime depression comorbidity were excluded from analyses. In both groups, SMC and test performances were not related but in BPD patients SMC were related to BPD symptoms.

**Conclusions:**

Our data indicate memory impairment of BPD patients in everyday life. However, it cannot be ruled out that increased memory complaints result from patients’ negative self-perception. Future research needs to clarify the reasons for memory complaints of BPD patients.

## Background

Patients with Borderline Personality Disorder (BPD) show a wide range of psychopathological symptoms such as instability, impulsivity, suicidal or self-harming behavior, and rage [1]. BPD patients also suffer from psychotic and dissociative symptoms, with disturbances of perception and of cognition, including memory [2-4]. However, studies that focused on neuropsychological tests revealed conflicting results. Memory dysfunctions were reported by some (e.g., [5]) but not all studies (e.g., [6]). Divergent results may be related to moderators. In some studies memory dysfunction of BPD patients was limited to dissociative patients [7], patients with lower education [8], or to experimental conditions that required the inhibition of emotionally negative distracters [9].

Apart from the administration of “objective” verbal memory tests, Park et al. [10] also asked their participants about everyday memory functioning. Participants came from a representative sample of US citizens. In this sample, BPD symptoms were associated with the subjective ratings but not with verbal memory test scores. Therefore, it seems possible that memory dysfunction in BPD is restricted to everyday life conditions and is not detectable in experimental settings. However, Park et al. did not apply a standardized questionnaire to assess subjective memory complaints (SMC) and participants were not assessed for axis I or axis II disorders. Furthermore, results were restricted to the verbal memory domain. Whereas, to our knowledge, studies with BPD patients are missing, SMC have been extensively investigated in subjects with other conditions. These studies indicate that SMC are related to clinical factors such as depression (e.g., [11]), anxiety (e.g., [12]), and neuroticism (e.g., [13]). Although the validity of SMC has sometimes been questioned [14], associations with brain measures [15-17] substantiate that SMC reflect real neurocognitive deficits. In addition, in a former study we verified the self-rating results with observer ratings and objective memory test outcome [18].

On the basis of the Park et al. study and studies with patients with similar disorders, such as MDD, we hypothesize that BPD patients report more memory problems than healthy subjects (main hypothesis). In addition, we hypothesize that SMC are related to BPD symptomatology, and, to a weaker extent, to memory test performance.

## Methods

### Participants

Based on earlier studies (e.g., [18]), we assumed a large effect for the main hypothesis. With a power analysis (g-power) we calculated a minimum of 28 subjects for each group, BPD patients and healthy control subjects, respectively (one-sided, *d* = 0.8, *alpha* = .05, *power* = .90). We then included 32 BPD patients and 32 healthy controls (HC). At the time of the study, all patients were treated as inpatients in a specialized ward for patients with BPD. First, BPD patients were screened by a clinical psychologist with respect to the inclusion and exclusion criteria. Secondly, a clinical psychologist and member of the research team (NR, CM, or KW) informed the respective patients and invited them to participate. After a clinical interview based on DSM IV criteria, we decided which patients to include in the study. Healthy subjects were recruited through advertisements (posters at the university, supermarkets, and libraries). Interested subjects were first screened through telephone interviews. Subjects who seemed to meet the inclusion criteria were invited and formally informed about the study. After interviewing them using the DSM IV criteria, we decided which subjects to include in the study.

Exclusion criteria for participation in the study included severe mental disorders (e.g., psychosis, anorexia, alcohol abuse or drug abuse) and severe physical disorders (e.g., neurological disorders involving the central nervous system, mental retardation, malignant diseases, and liver cirrhosis). In addition, pregnant women were not included. Healthy controls were free of any axis I or II disorders. We obtained written, informed consent from all the subjects. The study was approved by the IRB (University of Muenster Ethics Committee). Of note, data about memory test performance (but not about SMC) of some study participants have already been reported elsewhere [9,19]. For this reason, we decided to focus our hypotheses on SMC and their relation to memory test performance and BPD symptoms.

Age (BPD: 27.3, HC: 27.9), basic school education (BPD: 11.3 years, HC: 11.8 years), and gender (BPD: 66% women, HC: 65% women) were comparable in both groups and did not differ significantly (Table 1).

**Table 1:** **Means, standard deviations, effect sizes and other statistics of demographic data, subjective memory complaints, and memory test performance in healthy controls, BPD patients, and BPD patients without axis I and II comorbidity (BPD NOCOM)**

	**Healthy **	**BPD**	**BPD**	**Statistics**	**Statistics**
	**Controls**	**Patients**	**NOCOM**	**HC vs BPD**	**HC vs BPD NOCOM**
	**(n = 32)**	**(n = 32)**	**(n = 7)**		
**Demographic data**					
Age	27.2 (10.9)	27.3 (8.8)	30.6 (10.1)	t = 0.1, ns (d = 0)	t = 0.7, ns (d = 0.3)
Basic School Education (years)	11.4 (1.3)	11.3 (1.4)	11.6 (1.5)	t = 0.3, ns (d = 0.1)	t = 0.2, ns (d = 0.1)
Sex (f/m)	22/10	21/11	3/4	Chi^2^ = 0.1, ns (d = 0)	Chi^2^ = 1.7, ns (d = 0.4)
**SMC**					
EMQ	50 (13.5)	99 (37.5)	68 (19.2)	t = 7.0*** (d = 1.9)	t = 3.0** (d = 1.1)
**Memory tests**					
Logical Memory I	31 (7.7)	31 (7.0)	29 (4.5)	F = 0.2, ns (d = 0)	F = 0.5, ns (d = 0.3)
Logical Memory II	27 (8.0)	27 (8.1)	26 (6.0)	F = 0.0, ns (d = 0)	F = 0.1, ns (d = 0.1)
AVLT (round 1–3)	32 (4.6)	31 (5.2)	33 (6.8)	F = 2.2, ns (d = 0.2)	F = 0.2, ns (d = 0.2)
CFT (late recall)	21 (6.7)	19 (5.8)	21 (4.6)	F = 1.2, ns (d = 0.3)	F = 0.0, ns (d = 0)
RVDLT (round 1–3)	26 (7.1)	25 (7.3)	25 (7.2)	F = 0.3, ns (d = 0.1)	F = 0.0, ns (d = 0.1)

BPD = Borderline Personality Disorder, BPD NOCOM = BPD patients without axis I and II comorbidity, SMC = Subjective memory complaints, EMQ = Everyday Memory Questionnaire, AVLT = Auditory Verbal Learning Test, CFT = Complex Figure Test, RVDLT = Rey Visual Design Learning Test, d = Effect Size (all effect sizes are converted into Cohens d).

***p < .001, **p < .01, ns = non significant.

Several patients with BPD were noted to have comorbid mental disorders. Nineteen patients had evidence of anxiety disorders with some patients suffering from several anxiety disorders (posttraumatic stress disorder: n = 13, agoraphobia: n = 5, panic disorder: n = 3, social phobia: n = 2, obsessive compulsive disorder: n = 1, generalized anxiety disorder: n = 1, other anxiety disorder: n = 2). Fourteen patients suffered from affective disorders (major depression: n = 7, dysthymia: n = 7), 3 patients suffered from bulimia, and 1 patient had somatoform disorder. With regard to comorbid Axis II disorders, 11 patients fulfilled the diagnostic criteria for another personality disorder (avoidant: n = 6; dependent: n = 2; schizotypal, schizoid, paranoid: each n = 1). Seven patients were free of any current Axis I or comorbid Axis II disorder. Of the patients, 17 took antidepressants (SSRIs, SNARIs or SNRIs: n = 11, tricyclics: n = 4, lithium: n = 1, other antidepressants: n = 1). For sedation, 8 patients were receiving neuroleptics, 1 received antiepileptics, and 2 received benzodiazepines. Two patients were receiving pain medication, and 4 patients received antihypertensives. Eight patients were free of any medication. Apart from one person who received antihypertensives, none of the other healthy subjects took medication.

As expected, BPD patients reported much more BPD-related symptoms than healthy subjects (Borderline Symptom List [BSL]; [20]; Scores: BPD: 161 [82], HC: 31 [29]; (*t*_(76)_ = 9.8, *p* < .001). A mean BSL score of 161 (*SD* = 82) for the BPD patients indicates a moderate manifestation of BPD-related symptoms [20].

### Instruments

#### Clinical examination

Psychiatric diagnoses were made using the Structured Clinical Interviews for DSM IV, SCID-I for Axis I disorders, and SCID-II for personality disorders. These interviews were applied by trained psychotherapists. The clinical examination also included the assessment of BPD-related symptoms (Borderline Symptomlist [BSL]; [20]).

#### Memory tests

##### Verbal memory

*Auditory Verbal Learning Test (AVLT*; [21,22]): Subjects had to recall 15 simple words that were presented three (instead of five) times. Each learning trial was followed by an immediate free recall. Total score (max. 45 words) was used for subsequent analyses.

*Wechsler Memory Scale – Revised (WMS-R), Subtest Logical Memory* [23,24]: Subjects had to recall two short stories as accurately as possible. Recall performance was assessed immediately after each story was heard (logical memory I) and after twenty minutes (logical memory II). Total scores of each recall (max. 25 thematic units) were used for subsequent analyses.

##### Nonverbal memory

*Rey Visual Design Learning Test* (*RVDLT*; [25]): Subjects had to recall 15 simple designs that were presented three (instead of five) times. Each learning trial was followed by an immediate free recall. Total score (max. 45 designs) was used for subsequent analyses.

*Complex Figure Test* (*CFT;* [26])*:* Subjects had to recall and draw a complex figure, which they had previously been shown and had copied 30 minutes before. Total score (max. 36 details) was used for subsequent analyses.

#### Subjective memory complaints (SMC)

*Everyday Memory Questionnaire – 28* (*EMQ*, [27]): Subjects had to answer 28 questions with respect to the frequency of everyday memory problems (from 1 = not at all in the last six months to 9 = more than once a day). The items were related to several aspects of everyday life such as language (e.g., “Finding that a word is ‘on the tip of your tongue.’ You know what it is but cannot quite find it.”), nonverbal information (e.g., “Forgetting where things are normally kept or looking for them in the wrong way.”), or actions (e.g., “Forgetting details of things you do regularly, whether at home or at work. For example, forgetting details of what to do, or forgetting at what time to do it.”). Based on a principal component analysis, however, Sunderland et al. [27] regard the EMQ as a measure of everyday memory in general. We found that Chronbach’s Alpha and split half reliability were highly satisfying for the BPD patient group (Alpha = .94, Split Half = .94) and for the healthy control group (Alpha = .91, Split Half = .86).

### Statistical analyses

All statistical analyses were performed using the “Statistical Package for the Social Sciences 20 (SPSS 20).” Level of significance was set to *p* < .05 for all analyses and was two-tailed. Demographic data were analyzed by two-tailed t-tests or chi^2^-tests. We compared BPD patients’ and healthy participants’ SMC performance as assessed by means of the EMQ with a t-test. To control for comorbidity, in an exploratory analysis we also compared BPD patients without acute axis I and axis II comorbidities and healthy subjects. Because of the small sample size of the subsample of BPD patients without comorbidity (*n* = 7), we checked the respective results by non-parametric tests (Mann-Whitney-U-Test). Memory test performance of BPD patients and healthy participants was compared using a Multifactorial Analyses of Variance (MANOVA). Verbal (AVLT, Logical Memory I and II of the WMS-R) and nonverbal memory test results (RVDLT, CFT) served as dependent variables. To control for comorbidity, in an exploratory analysis we also compared BPD patients without acute axis I and axis II comorbidities and healthy subjects. Again, because of the small sample size of the subsample of BPD patients without comorbidity, we checked the respective results by non-parametric tests (Mann–Whitney-U-Test). To explore the predictive value of BPD symptoms (BSL scores) and the memory test performance on SMC in BPD patients and healthy participants, we first conducted correlations between these variables. Because we administered 5 different memory tests we applied the Bonferroni-Holm Correction [28] for the correlations between SMC and each memory test (*p* = .01). All other correlations in Table 2 were not related to our hypothesis and, therefore, not subject to the Bonferroni-Holm Correction. Secondly, we considered those variables that were correlated with SMC as predictors in regression analyses step by step (method enter).

**Table 2:** **Correlations between subjective memory complaints as assessed by the Everyday Memory Questionnaire (EMQ), Borderline Symptom List (BSL), comorbidity (COM), and memory test performance in patients with Borderline Personality Disorder (n = 32, numbers above the diagonal) and healthy participants (n = 32, numbers below the diagonal)**

	**EMQ**	**BSL**	**COM**	**AVLT**	**Log I**	**Log II**	**RVDLT**	**CFT**
EMQ	-	0.54**	0.47*	−0.08	−0.02	−0.03	−0.18	0.01
BSL	0.41*	-	.55**	−0.19	0.13	0.04	−0.06	−0.01
COM	-	-	--	−0.21	0.10	0.08	0.00	−0.14
AVLT	0.14	0.08	-	-	0.40*	0.28	0.48**	0.39*
Log I	0.20	0.10	-	0.33	-	0.92***	0.57**	0.09
Log II	0.16	0.03	-	0.26	0.94***	-	0.49**	0.10
RVDLT	0.44*^1^	0.34	-	0.26	0.20	0.11	-	0.28
CFT	0.25	0.17	-	0.56**	0.13	0.10	0.53**	-

EMQ = Everyday Memory Questionnaire, BSL = Borderline Symptom List, AVLT = Auditory Verbal Learning Test, Log I = Logical memory immediate recall, Log II = Logical memory delayed recall, RVDLT = Rey Visual Design Learning Test, CFT = Complex Figure Test.

***p < .001, **p < .01, *p < .05, ^1^This correlation failed the adjusted level of significance (alpha = 0.01).

## Results

### Subjective memory complaints (SMC)

BPD patients showed more subjective memory complaints than healthy subjects (Table 1). To evaluate the impact of comorbid disorders, in an exploratory analysis we compared healthy subjects to BPD patients without acute axis I and axis II comorbidity (*n* = 7). The effect remained stable (Table 1) even when the Mann-Whitney-U-Test was applied (SMC as assessed by the EMQ: *Z* = 2.4, *p* = 0.16). Effect sizes (Table 1) indicate a clinically relevant impairment in the BPD patients (*d* = 1.9).

### Memory test performance

Memory test performance of BPD patients and healthy participants were comparable (*F*_(5, 88)_ = 0.6, *p* = 0.67) even when univariate analyses were applied (Table 1). In an exploratory analysis we compared healthy subjects to BPD patients without acute axis I and axis II comorbidity (*n* = 7), and these results also remained stable (*F*_(5, 63)_ = 0.3, *p* = 0.91; for univariate analyses see Table 1). These results were confirmed by separate Mann-Whitney-U-Tests: No group differences were revealed with respect to memory tests (all *p* > .28). Effect sizes confirmed that the BPD patients’ memory test performance was not relevantly impaired (all *d* < 0.4, see Table 1).

### Prediction of subjective memory complaints (SMC) by BPD symptoms and memory test performance

Correlation analyses indicated that SMC are related to BPD symptoms and to the presence of comorbid disorders (yes versus no) in the BPD group, but SMC are not related to memory test performance (Table 2). In the subsequent regression analyses, at first we only included BPD symptoms to predict SMC because BPD symptoms showed the highest correlation with SMC. With an explained variance of 29%, the model reached statistical significance (*F*_(1, 21)_ = 8.8, *p* = 0.007). For the next step we also included comorbidity. Explained variance increased to 34%, however the increase of explained variance failed the level of significance (*F*_(1, 20)_ = 1.5, *p* = 0.24).

In healthy participants, correlation analyses indicated that SMC are only related to BPD symptoms. For explorative purposes, in the subsequent regression analyses we also considered visual learning as assessed by means of the Rey Visual Design Learning Test (Table 2). There was a statistically significant relationship between visual learning and SMC if alpha was not corrected. In the first step, however, we only included BPD symptoms in order to predict SMC as BPD symptoms. With 17% explained variance, the model reached statistical significance (*F*_(1, 29)_ = 5.7, *p* = 0.023). In the next step, we also included the delayed recall of verbal information. Explained variance increased to 27%, however this increase failed the level of significance (*F*_(1, 28)_ = 3.8, *p* = 0.06).

## Discussion

To our knowledge, everyday memory functioning of BPD patients has never been systematically investigated. In the present study, we asked BPD patients and healthy participants about subjective memory complaints (SMC) by means of the Everyday Memory Questionnaire [27]. In addition, we administered verbal and nonverbal memory tests. While the patients’ test performance was normal, they reported memory problems in their everyday life. These problems were related to BPD symptoms but not to memory test performance.

Our finding of increased SMC in the BPD group is in agreement with Park et al. [10], who found an association between SMC and borderline symptoms in a representative sample of US citizens. As symptoms of depression and anxiety are typical for BPD patients, the findings of the present study are also supported by studies that found associations between SMC and depression as well as between SMC and anxiety [11,12]. Anxiety disorders and depression are also frequent comorbid disorders of BPD [29]. Along these lines, comorbidity in our study (namely anxiety and affective disorders) was positively correlated with BPD symptoms and SMC. However, comorbidity did not explain SMC variance beyond BPD symptoms and BPD patients without comorbid axis I or other axis II disorders also showed more SMC than healthy participants. These results indicate, first, that our study results are not due to the comorbid disorders of the BPD patients. Second, these results indicate that additional DSM IV diagnoses are not of additional value in predicting SMC in BPD patients given the great overlap between BPD symptoms and anxiety as well as between BPD symptoms and depression. However, SMC may be related to commonalities among these disorders such as emotion regulation difficulties. Third, the study results indicate that everyday memory problems increase with an increase in the severity of the disorder.

SMC were not related to memory test performance in the patient group. This result is in agreement with the already mentioned Park et al. study [10] and the result was also found in different patient groups such as MDD patients (e.g., [30]). Other studies, however, found associations between SMC and memory tests in MDD patients, but these associations were described as being modest [18]. The missing correlation between SMC and memory test results in the present study can be explained by differences between the demands of everyday life and the test setting. That is, in a test setting tasks are well circumscribed. All disturbing and distracting influences are excluded and the subject’s performance is continuously monitored by the examiner. By contrast, the requirements of everyday life are much less structured and frequently more complex than the requirements of psychometric tests. These requirements often have to be self-organized and self-paced and are susceptible to various kinds of disturbances, including emotionally relevant distracters. In a recent study we found that (verbal) memory performance of BPD patients was normal when no distracters were present or when emotionally neutral distracters were present [9]. By contrast, the patients’ performance was impaired when emotionally negative distracters were displayed. If possible, future research should investigate these factors in real-life situations.

However, it is also possible that the self-reported, increased SMC of BPD patients are influenced by the patients’ biased self-perception. A biased self-perception in BPD patients was found in a recent study [31]. The authors assessed retrospective ratings of specific emotions in patients with BPD and in healthy controls. While healthy controls showed a positive recall pattern, BPD patients showed an overall negative recall pattern. Another study revealed that BPD patients’ negative self-perception is similar to that of MDD patients [32]. In MDD, a negatively biased self-perception has been known for many years [33]. From this point of view, correlations between SMC and BDP symptoms could be explained by the assumption that the results of both respective self-rating scales used here are biased by biased self-perception. Alternatively, a biased self-perception may be greater in patients with more severe symptoms. Biased self-perception could also explain missing correlations between SMC and memory tests. In contrast to this assumption, in healthy participants correlations between SMC and memory tests also failed to reach the level of significance and were numerically small. However, future studies should consider the assessment of biased self-perception in order to control for its influence on SMC.

What can be concluded from the study results? First, BPD patients may suffer from cognitive impairments in everyday life even when neuropsychological test results are normal. Neuropsychological assessment of BPD patients should, therefore, include the administration of questionnaires that are related to everyday cognitive functioning. If there is sufficient evidence for everyday related cognitive dysfunctions in individuals with BPD, then these dysfunctions should be treated. It is known that untreated cognitive impairments in patients with mental disorders are associated with reduced level of psychosocial and occupational functioning [34], reduced compliance [35] and risk of suicide [36]. Secondly, increased complaints about cognitive functioning in everyday life are diagnostic criteria in MDD. It could be concluded from the present study that these criteria might also be acknowledged in the diagnosis of BPD. Of course, further research has to confirm the present findings. It would also be helpful to consider different neuropsychological domains apart from memory functioning, such as attention and executive functioning.

The major shortcoming of the present study is the questioned validity of SMC questionnaires. As pointed out above, the results of the present study may also reflect the negatively biased self-perception of BPD patients, especially as the items of the Everyday Memory Questionnaire (EMQ) are uniformly phrased (with 1 = no indication of memory problems and 9 = indication of the most severe memory problems). However, the validity of the EMQ has been widely demonstrated [37]. In addition, it has been shown that self-reported cognitive deficits of mentally ill patients, in general, cannot be exclusively explained by response biases [15-18]. Another limitation of the present study concerns the comorbid disorders of the BPD patients investigated. It is currently unclear how to manage these multiple comorbidities. Given the high comorbidity rates in BPD, the exclusion of these patients may lead to a study sample that is not representative of the general population. Conversely, comorbid disorders that impact the variables tested may have influenced the study results. However, we repeated the main statistical analyses for a subgroup of BPD patients without acute mental disorders and without further personality disorders and found similar results. These results have to be regarded as preliminary because the sample size of this subgroup (*n* = 7) is very small. As the clinical interview administered in the present study (Structured Clinical Interview for DSM IV) does not screen for all mental disorders, the influence of these disorders (e.g., ADHD) on the study results cannot be ruled out.

## Conclusions

In conclusion, BPD patients reported memory problems in their everyday life but did not show impairments in memory tests. These results support the notion that neuropsychological assessment of BPD patients should consider everyday related cognitive functioning apart from the administration of tests. If patients show evidence of impaired test performance or everyday related cognitive dysfunctions, then they may profit from neuropsychological therapy. To control for the influence of biased self-perception on self-reported cognitive dysfunction, future studies should include other techniques to assess cognitive functioning in everyday life such as observer ratings and the systematic analyses of real-life situations. In addition, self-perception itself should also be assessed.
